# Linking Degradation Pathways, Additive Transformation, and Contaminant Profiles in Post-Consumer HDPE: Implications for Recycling Quality

**DOI:** 10.3390/polym18111369

**Published:** 2026-05-31

**Authors:** Marek Kucbel, Helena Raclavská, Jana Růžičková, Michal Šafář, Barbora Švédová, Karolina Slamová, Pavel Kantor, Petr Braun

**Affiliations:** 1 ENET Centre, Centre for Energy and Environmental Technologies, VSB–Technical University of Ostrava, 17. listopadu 15/2172, 708 00 Ostrava-Poruba, Czech Republic; helena.raclavska@vsb.cz (H.R.); jana.ruzickova@vsb.cz (J.R.); michal.safar@vsb.cz (M.Š.); barbora.svedova@vsb.cz (B.Š.); pavel.kantor@vsb.cz (P.K.); 2 Institute of Foreign Languages, VSB–Technical University of Ostrava, 17. listopadu 15/2172, 708 00 Ostrava-Poruba, Czech Republic; karolina.slamova@vsb.cz; 3 TESO, Urbánkova 3367, 143 00 Prague, Czech Republic; braun.teso@gmail.com

**Keywords:** high-density polyethylene (HDPE), post-consumer plastics, chemical recycling, pyrolysis–GC/MS, polymer degradation, recycling quality

## Abstract

The chemical complexity of post-consumer plastics represents a major challenge for achieving high-quality recycling. In this study, post-consumer high-density polyethylene (HDPE) packaging materials were analysed using pyrolysis–gas chromatography–mass spectrometry (Py-GC/MS) to investigate relationships between compound origin, degradation pathways, and contaminant profiles. More than one hundred organic compounds were detected and classified into four main groups: product-related inputs, polymer formulation chemistry, polymer degradation processes, and external contamination. Polymer degradation products, particularly radical rearrangement and cyclisation compounds, represented the most diverse group, indicating advanced transformation of the polymer matrix associated with repeated processing. Additive-derived compounds, including phenolic structures and epoxide-containing species, contributed to the pool of non-intentionally added substances (NIAS), while persistent compounds, such as fluoropolymer-derived residues, were detected across most samples. In contrast, product-related inputs showed high variability and a generally lower contribution. Multivariate analysis revealed that samples were not clustered according to product category but rather distributed along gradients defined by degradation, additive transformation, and contamination processes. These findings demonstrate that the chemical composition of recycled HDPE is determined or influenced by multiple independent factors. The results support the need for chemistry-informed recycling strategies.

## 1. Introduction

The transition toward a circular plastics economy requires not only increased recycling rates but also improved quality and safety of recycled materials. Among polyolefins, high-density polyethylene (HDPE) is one of the most widely used polymers in packaging applications due to its chemical resistance, mechanical strength, and low permeability [1,2,3]. Consequently, post-consumer HDPE represents a major fraction of plastic waste streams and is increasingly considered for higher-value recycling applications, including closed-loop systems [4].

However, the quality of recycled HDPE (rHDPE) is frequently compromised by chemical degradation and the accumulation of low-molecular-weight organic compounds throughout the material lifecycle. These compounds originate from multiple sources, including residues of packaged products such as detergents, cosmetics, and fragrances [5,6,7], additives incorporated into the polymer matrix [8,9], and degradation products formed during processing and use [10,11]. Their presence can adversely affect mechanical performance, odour, and regulatory compliance, thereby limiting the applicability of recycled materials [2,12]. Recent data-driven approaches have further highlighted the variability and complexity of post-consumer HDPE recyclates and their implications for high-value applications such as bottle-to-bottle recycling [13].

A key factor governing the persistence of these compounds is their interaction with the semi-crystalline structure of polyolefins. HDPE consists of crystalline domains embedded in an amorphous phase, which enables diffusion and sorption of organic molecules [10,14]. This results in the so-called “memory effect”, in which compounds originating from previous contents remain embedded in the polymer matrix even after extensive washing [12,15]. Low-polar and lipophilic substances, such as terpenes, aromatic esters, and ketones, exhibit particularly strong affinity to polyolefins and are therefore difficult to remove during conventional recycling processes [12,16]. In addition, recent work on non-intentionally added substances (NIASs) highlights the complexity of chemical mixtures present in recycled plastics and the associated analytical challenges [17].

In parallel, HDPE undergoes thermo-oxidative degradation during repeated processing and use. This process proceeds via radical chain reactions involving hydroperoxide formation and decomposition, leading to the formation of oxygenated compounds such as aldehydes, ketones, and alcohols, as well as hydrocarbons formed through β-scission of the polymer backbone [10,18]. The influence of thermal and thermo-oxidative conditions on the chemical and structural evolution of HDPE has been extensively studied [19], including under long-term environmental and accelerated ageing conditions [20]. Under more advanced conditions, secondary reactions such as cyclisation and aromatisation may lead to the formation of cyclic and aromatic hydrocarbons [1,8].

Despite extensive research, contamination and degradation in recycled plastics are typically investigated separately. Most studies focus either on the degradation mechanisms of polyolefins [10,18,19] or on specific groups of contaminants and their migration behaviour [12,16,17], without establishing a systematic relationship between compound origin, chemical composition, and degradation pathways. As a result, the interpretation of complex analytical datasets derived from real post-consumer materials remains limited, and robust chemical indicators of recyclate quality remain insufficiently defined, despite recent advances [21].

This study addresses this gap by establishing a direct relationship between the origin of compounds, degradation pathways, and persistence in real post-consumer HDPE systems. By integrating chemical profiling with mechanistic interpretation, the work enables differentiation between product-derived, polymer-derived, and degradation-related compounds within complex material matrices. This represents a shift from conventional compositional analysis toward a more systematic understanding of how different chemical processes jointly define material composition and quality.

This study aims to establish a systematic relationship between chemical composition, compound origin, and degradation pathways in post-consumer HDPE and to evaluate their combined relevance for quality assessment of recycling. Specifically, the study: (i) identifies and classifies organic compounds using Py-GC/MS; (ii) distinguishes between compounds originating from polymer degradation, additives, and residual contents; and (iii) evaluates their relevance as indicators of material quality in recycling processes.

This study contributes to bridging the gap between controlled laboratory studies and the complex chemical reality of post-consumer plastic materials by providing a comprehensive characterisation of real-world HDPE packaging samples.

## 2. Materials and Methods

### 2.1. Sample Preparation and Classification

A total of 18 post-consumer HDPE packaging samples were analysed and classified into five application groups according to the type of original content: (i) Hard surface cleaners: Duck, Domestos, Bref WC, Cif spray, Cif cream, Jar; (ii) Laundry detergents: Persil, Denk mit, Denk mit Color, Vanish; (iii) Cosmetic products: Palmolive shower, Nivea shower, Manufactura shower; (iv) Fragrances/fabric softeners: Lenor, Tesori d’Oriente, Muschio Bianco; (v) Pharmaceutical products: Omeprazole.

The pharmaceutical packaging sample (Omeprazole) was included as a low-interference reference system, as it is not associated with lipid-rich or fragrance-based product formulations. This sample was used as a baseline to distinguish between product-derived and polymer-related compounds. Before analysis, all packaging samples were subjected to a standardised rinsing procedure comprising ten consecutive cycles of filling with water, followed by complete emptying. This procedure may lead to partial removal of highly polar and low-molecular-weight compounds; however, its uniform application ensures comparability between samples.

This procedure was applied to remove freely present product residues while preserving compounds sorbed within the polymer matrix. Labels were removed and analysed separately where applicable. The packaging materials were mechanically reduced to fragments smaller than 5 mm before analysis.

### 2.2. Pyrolysis–Gas Chromatography–Mass Spectrometry (Py-GC/MS)

Chemical characterisation was performed using pyrolysis–gas chromatography–mass spectrometry (Py-GC/MS). Pyrolysis-GC/MS analysis was performed using a Frontier AS-2020E Auto-Shot Sampler/pyrolysis system (Frontier Laboratories Ltd., Koriyama, Fukushima, Japan) coupled to an Agilent 5977C GC/MSD system (Agilent Technologies, Inc., Santa Clara, CA, USA). Approximately 100–500 µg of each sample was placed into a metal sample cup together with a small amount of glass wool, which was used to stabilise the sample within the pyrolysis cup, ensure consistent positioning during heating, prevent sample loss due to rapid volatilisation, and promote more uniform heat distribution during pyrolysis. The samples were subjected to single-shot pyrolysis at 600 °C for 60 s. These conditions were selected to ensure reproducible thermal fragmentation of polyethylene and comparable pyrograms across all samples.

The resulting pyrolysates were introduced into the GC system via split injection (split ratio 1:500) with an inlet temperature of 300 °C. Separation was performed on a non-polar metal capillary column Ultra ALLOY^®^ UA+Py2 (Frontier Laboratories Ltd., Koriyama, Fukushima, Japan) 30 m × 0.25 mm × 0.50 µm. The oven temperature program was as follows: 40 °C (2 min), ramped to 320 °C at 20 °C/min, then held at 320 °C for 5 min.

Mass spectra were acquired using the MS detector and evaluated using library-based identification F-Search software, version 3.8 (Frontier Laboratories Ltd., Koriyama, Fukushima, Japan). Compound identification was based on comparison with spectral databases (e.g., NIST) and interpreted with consideration of known pyrolysis products of polyethylene, additives, and related materials.

Identified compounds are reported using names corresponding to GC-MS library databases. These names may not fully conform to systematic IUPAC nomenclature, but allow the consistent and unambiguous identification within the context of Py-GC/MS analysis. All measurements were performed in duplicate. The reported values represent average relative abundances, with variability between replicate analyses generally below 7%.

The analysis was performed using a semi-quantitative Py-GC/MS fingerprinting approach, and results are reported as relative peak area percentages. This approach is primarily intended for the comparative evaluation of chemical profiles across samples rather than absolute quantification; it relies on the high reproducibility of Py-GC/MS under controlled conditions.

The selected pyrolysis temperature represents a compromise between efficient depolymerisation and preservation of structurally informative degradation products. While secondary reactions such as cracking, cyclisation, and aromatisation may occur at elevated temperatures, these products provide relevant information on degradation pathways rather than being treated as analytical artefacts. In this context, Py-GC/MS provides information on volatile degradation products, whereas complementary techniques such as thermogravimetric analysis (TGA) are more suitable for assessing bulk thermal behaviour and residual mass formation.

### 2.3. Data Processing and Semi-Quantitative Evaluation

The relative abundances of individual compounds were estimated from their peak areas in the chromatograms. The obtained values are semi-quantitative and do not represent absolute concentrations. Therefore, all interpretations are based on relative comparisons between samples.

### 2.4. Semi-Quantitative Degradation Index

A semi-quantitative degradation index (DI) was defined to estimate the relative extent of polymer degradation in individual samples. The index was based on grouped contributions of compounds associated with specific degradation pathways, including: (i) primary oxidation products; (ii) secondary oxidation products; (iii) radical rearrangement and cyclisation products; (iv) thermal degradation products (alkenes and dienes); and (v) aromatisation products.

Each group was assigned a weighting factor reflecting its relative position within the degradation pathway and its association with increasing structural modification of the polymer matrix. Primary oxidation products were assigned a weight of 1, as they represent early-stage oxidation processes. Secondary oxidation products were assigned a weight of 2, reflecting further progression of oxidative degradation. Thermal degradation products (alkenes and dienes) were also assigned a weight of 2, as they indicate chain scission processes without extensive structural rearrangement. Radical rearrangement and cyclisation products were assigned a weight of 3, as they represent advanced degradation involving radical recombination and structural transformation. Similarly, aromatisation products were assigned a weight of 3, reflecting extensive structural modification associated with the formation of cyclic and aromatic systems.

The degradation index was calculated as the sum of weighted relative abundances of the individual groups. The resulting values provide a comparative measure of degradation intensity across samples rather than an absolute quantification of degradation.

The weighting factors were defined based on the relative importance of individual compound groups in describing the degradation state of the material, considering both their mechanistic relevance and their contribution to variability in the dataset. The DI is intended as a semi-quantitative metric for comparative assessment rather than absolute quantification.

### 2.5. Multivariate Analysis (PCA)

Principal component analysis (PCA) was performed to explore relationships between degradation processes, additive-derived compounds, and contaminant profiles across samples. To reduce data complexity, individual compounds were grouped into aggregated chemical categories based on their origin and functional relevance: (i) degradation-related compounds; (ii) additive-derived compounds (including stabilisers and NIAS); (iii) polymer-related lipid and wax-like structures; (iv) plasticisers and related compounds, (v) fluoropolymer-derived residues; and (vi) product-derived compounds. For each sample, the relative abundances within each category were summed. Missing values were treated as zero, reflecting the absence of detectable compounds. Before PCA, the data were standardised using autoscaling (mean-centring and unit-variance scaling). PCA was performed on the scaled data using the covariance matrix in Python 3.14 software environment using the pandas 2.3, NumPy 2.1, scikit-learn 1.7, and Matplotlib 3.9 libraries (Python Software Foundation, Wilmington, DE, USA). The first two principal components were used for interpretation.

## 3. Results

### 3.1. Overview of Identified Compounds

**Figure 1:**
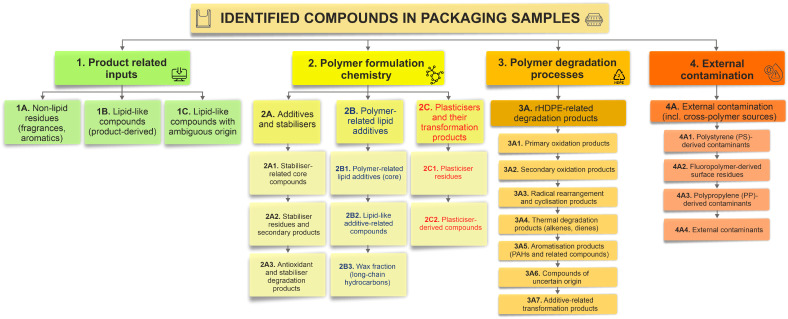
Classification of compounds identified in post-consumer HDPE according to their origin and formation pathways.

A total of 137 compounds were identified across all packaging samples and classified into four major groups based on their origin and functional relevance: product-related inputs, polymer formulation chemistry, polymer degradation processes, and external contamination (Figure 1).

Among these, polymer degradation processes represented the most diverse group, comprising the highest number of unique compounds. This category was dominated by radical rearrangement and cyclisation products (n = 29), followed by aromatisation products (n = 8) and oxidation-derived compounds. In contrast, thermal degradation products were comparatively limited (n = 3), indicating a minor contribution of high-temperature degradation pathways.

Polymer formulation chemistry also contributed substantially to the overall diversity of compounds. Within this group, polymer-related lipid additives (core compounds) showed high diversity (n = 13), followed by lipid-like additive-related compounds (n = 10). Stabiliser-related compounds were present but less diverse overall, particularly for core stabilisers (n = 7) and their degradation products. Product-related inputs comprised three distinct subgroups: non-lipid residues (fragrance- and aroma-related compounds; n = 15), lipid-like product-derived compounds (n = 4), and lipid-like compounds of ambiguous origin (n = 7).

External contamination represented the least diverse group, with contributions primarily from polystyrene-derived contaminants (n = 8) and fluoropolymer-derived surface residues (n = 4), while polypropylene-derived and other external contaminants were sporadically detected.

**Figure 2:**
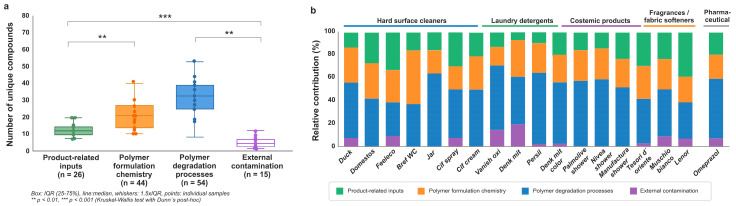
Polymer formulation-related compounds in post-consumer HDPE; (**a**) Number of unique compounds per formulation-related subgroup; (**b**) Relative contribution (%) of major formulation-related groups across product categories. Detailed distribution of compound subgroups across individual samples is shown in Supplementary Figure S1.

The overall distribution of compound diversity and the relative contribution of the four main groups are summarised in Figure 2. The boxplot (Figure 2a) shows the number of unique compounds per group, with the highest diversity for polymer degradation processes and the lowest for external contamination. The stacked bar chart (Figure 2b) presents the relative contribution of the main groups across individual samples and product categories. The heatmap (Supplementary Figure S1) provides a detailed overview of the distribution of compound subgroups across all samples, highlighting the internal structure of the classification system.

The pharmaceutical sample (Omeprazole) showed a relatively high contribution from lipid-like compounds of ambiguous origin compared to other samples.

In summary, the chemical profile of the analysed packaging samples was predominantly shaped by polymer degradation processes and formulation-related compounds, while product-related inputs and external contamination contributed to a lesser extent.

### 3.2. Product-Related Inputs

**Figure 3:**
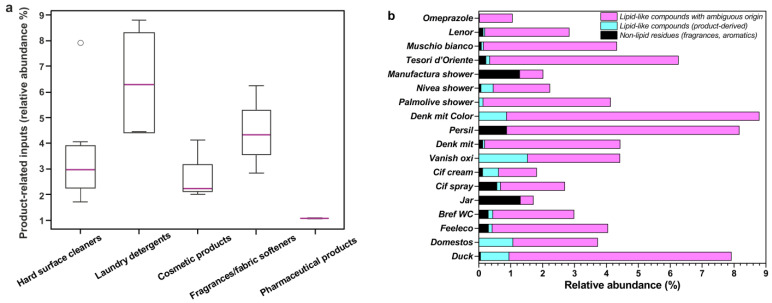
Distribution of product-related inputs across samples; (**a**) Relative abundance across product categories; (**b**) Composition of product-related inputs at the sample level.

Figure 3 presents the distribution of product-related inputs across product categories, with variations in relative abundance shown in Figure 3a. A complete overview of all identified compounds, including their classification, occurrence across samples, and associated statistical parameters, is provided in Supplementary Table S1. The highest values were observed in laundry detergent samples, while lower contributions were detected in hard surface cleaners and cosmetic products. Fragrance and fabric softener samples showed intermediate values, whereas the pharmaceutical sample exhibited the lowest contribution. The composition of product-related inputs at the sample level is presented in Figure 3b. Product-related inputs were further divided into three subgroups: non-lipid residues (fragrances and aromatics), lipid-like compounds derived directly from product formulations, and lipid-like compounds of ambiguous origin.

Non-lipid residues were primarily detected in samples associated with fragranced products and detergents, reflecting the presence of volatile and semi-volatile aromatic compounds typical of these formulations. Their contribution varied across samples, with higher values generally observed in laundry detergents and fabric softeners.

Lipid-like compounds directly associated with product formulations were identified in several samples, particularly in cosmetics and selected detergent products. However, their overall contribution remained relatively low compared to other compound groups.

In contrast, lipid-like compounds of ambiguous origin were among the most abundant compound groups across all samples, frequently exceeding the contribution of clearly assigned product-derived compounds (Figure 3b). These compounds were consistently detected across all product categories, including samples with minimal expected lipid input.

No clear relationship was observed between the abundance of lipid-like compounds with ambiguous origin and the relative contribution of degradation-related compounds. These compounds were also detected in the pharmaceutical packaging sample (Omeprazole), which exhibited no significant contribution from lipid-rich product formulations.

In summary, product-related inputs contributed differently to the chemical profiles of packaging materials across samples and product categories. Lipid-like compounds of ambiguous origin were consistently present and represented a substantial fraction of the detected compounds.

### 3.3. Polymer Formulation Chemistry

Polymer formulation chemistry contributed substantially to the overall chemical profile of the analysed packaging samples, both in terms of compound diversity and relative abundance (Figure 2b and Supplementary Figure S1). A comprehensive overview of these compounds, including stabilisers, lipid additives, and plasticisers, along with their transformation products, is provided in Supplementary Table S2, including their classification and occurrence across samples. 

Stabiliser-related compounds were detected only in selected samples and showed a highly uneven distribution. Core stabiliser structures were identified in a limited number of cases, with relatively high abundance in specific samples (e.g., Jar: 2.17%, Manufactura shower: 1.44%), while being absent in most others. Stabiliser residues and secondary products were more broadly distributed, with notable contributions in Bref WC (2.99%) and Tesori d’Oriente (1.31%), but generally remained at low levels in most samples. Antioxidant and stabiliser degradation products were detected across multiple product categories, particularly in hard surface cleaners (e.g., Domestos: 0.52%, Bref WC: 0.57%) and cosmetic samples (Nivea shower: 0.67%, Manufactura shower: 0.39%). Several identified compounds corresponded to phenolic structures, including biphenyl and substituted phenolic derivatives.

Polymer-related lipid additives constituted the most prominent and consistently detected subgroup within this category. The total contribution of this subgroup varied markedly across samples, ranging from negligible levels (e.g., Jar: 0.045%; Lenor: 0.024%) to higher values, which were observed particularly in cleaning and detergent products (e.g., Domestos: 4.34%; Cif cream: 5.71%; Muschio Bianco: 3.93%). Core lipid additives were present across nearly all samples, with elevated abundances in Domestos (2.31%) and Cif cream (3.40%). Lipid-like additive-related compounds showed similarly widespread occurrence, with higher values in Persil (1.53%), Palmolive shower (1.46%), and Nivea shower (1.22%). The wax fraction (long-chain hydrocarbons) was also consistently detected, with notable contributions in Cif spray (1.93%), Cif cream (2.02%), and Muschio Bianco (2.49%). Measurable levels of polymer-related lipids were also detected in the pharmaceutical packaging sample (Omeprazole: 0.97%).

Plasticisers and their transformation products were a minor, highly sample-specific component of polymer formulation chemistry. Detectable levels were observed only in selected samples, including Duck (0.63%), Vanish Oxi (0.31%), Denk mit Color (0.42%), and several cosmetic and fragrance-related products (e.g., Nivea shower: 0.04%, Manufactura shower: 0.27%, Tesori d’Oriente: 0.17%), as well as in the pharmaceutical packaging sample (omeprazole: 0.26%). In contrast, plasticisers were absent in most of the samples.

**Figure 4:**
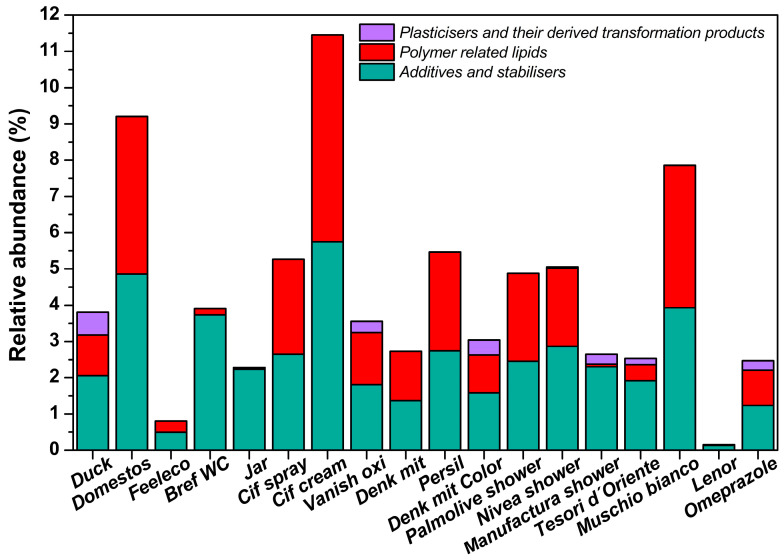
Polymer formulation chemistry across samples. Relative contribution (%) of stabilisers, polymer-related lipids, and plasticisers.

The relationship between polymer formulation chemistry and polymer degradation processes is shown in Figure 4. No clear relationship was observed between the total contribution of formulation-related compounds and the abundance of degradation products across the analysed samples.

In summary, polymer formulation chemistry showed variability across samples, with polymer-related lipid components consistently present, while stabilisers and plasticisers were more sample-specific.

### 3.4. Distribution and Classification of Polymer Degradation Products

Polymer degradation processes represented the most diverse group of compounds identified in the analysed packaging samples, comprising a total of 29 compounds associated with radical rearrangement and cyclisation pathways, alongside smaller groups of oxidation-derived and thermally induced products (Supplementary Figure S1).

To facilitate interpretation, the detected compounds were classified according to their formation pathways, including primary oxidation, secondary oxidation, radical rearrangement and cyclisation, thermal degradation, aromatisation, compounds of uncertain origin, and additive-related transformation products.

Radical rearrangement and cyclisation products constituted the dominant subgroup across the analysed samples, both in terms of diversity (n = 29) and relative abundance. These compounds were consistently detected at levels typically ranging from approximately 0.4% to 2.2% in individual samples, with higher contributions observed particularly in detergent- and cosmetic-related packaging.

Primary oxidation products were detected at relatively low levels (typically below 0.2%), while secondary oxidation products showed slightly higher but still moderate contributions (generally below ~1.7%). Thermal degradation products, including alkenes and dienes, were detected only sporadically and at very low levels (typically <0.2%). Similarly, aromatisation products (n = 8), including polycyclic aromatic hydrocarbons and related compounds, were present at low abundance (generally below ~1%).

A comprehensive overview of degradation-related compounds, including their classification into primary and secondary oxidation products, radical rearrangement structures, thermal degradation products, and aromatisation-derived compounds, together with their occurrence across the 18 analysed samples and associated statistical parameters, is provided in Supplementary Table S3. Compounds of uncertain origin and additive-related transformation products were detected in selected samples, with the latter showing localised contributions that occasionally reached approximately 2%.

The overall contribution of polymer degradation processes ranged between approximately 0.7% and 3.0% across samples, with notable variability between product categories. Laundry detergent packaging exhibited the widest range and highest values, whereas cosmetic products showed relatively consistent levels. Fragrance-related products generally displayed lower contributions, and the pharmaceutical packaging sample (omeprazole) exhibited one of the lowest overall degradation levels (~0.74%).

**Figure 5:**
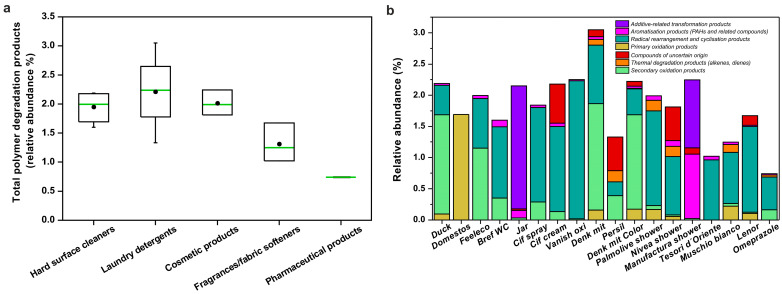
Polymer degradation processes in post-consumer HDPE; (**a**) Distribution of total degradation-related compounds across product categories; (**b**) Relative contribution (%) of compounds associated with different degradation pathways.

The distribution of polymer degradation contributions across product categories is summarised in Figure 5, which illustrates the variability within and between sample groups.

#### Degradation Index

A semi-quantitative degradation index (DI) was used to compare the overall extent of polymer degradation across the analysed packaging samples. The DI integrates contributions from different degradation pathways using weighted values that reflect their relative positions within the degradation sequence.

**Figure 6:**
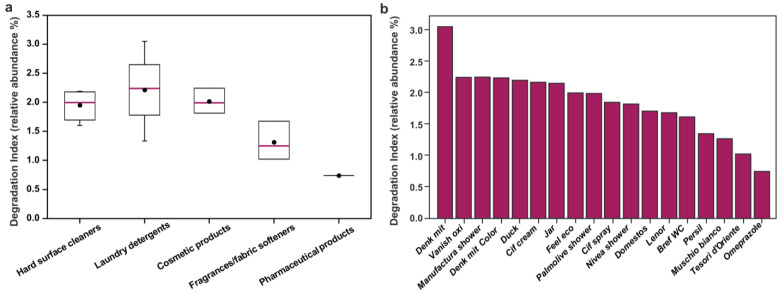
Degradation index (DI) of post-consumer HDPE samples; (**a**) Distribution of DI across product categories; (**b**) Samples ranked by DI values.

The calculated DI values revealed substantial variability between individual samples (Figure 6). The highest values were observed in detergent-related packaging, particularly in Denk mit and Vanish Oxi samples, which exhibited elevated contributions of radical rearrangement and secondary oxidation products.

Intermediate DI values were observed for most cosmetic products, which showed relatively consistent degradation levels across samples. Fragrance and fabric softener packaging generally exhibited lower DI values, although some variability remained. The lowest DI value was observed in the pharmaceutical packaging sample (Omeprazole). It showed minimal contributions from degradation-related compounds and served as a low-degradation reference within the dataset. In summary, the DI highlights differences in degradation intensity across product categories and provides a comparative metric for evaluating the relative extent of polymer degradation between samples.

### 3.5. External Contamination

**Figure 7:**
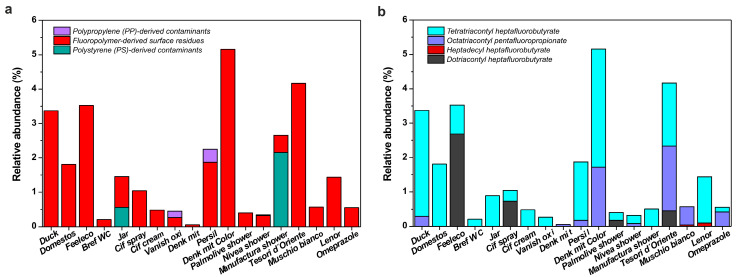
External contamination in post-consumer HDPE; (**a**) Relative contribution (%) of PS-, PP-, fluoropolymer-derived, and other contaminants; (**b**) Relative abundance of selected fluorinated compounds across samples.

The distribution of external contamination across individual samples is shown in Figure 7. External contamination represented a minor but clearly identifiable component of the detected chemical profiles, with contributions from polystyrene (PS)-, polypropylene (PP)-, and fluoropolymer-derived compounds. Among these, fluoropolymer-derived surface residues constituted the dominant subgroup and were detected in the majority of samples. Their relative abundance varied considerably, ranging from approximately 0.058% (Denk mit) to 5.153% (Denk mit Color), with elevated levels also observed in Duck (3.369%), Feeleco (3.520%), and Tesori d’Oriente (4.169%).

In contrast, PS-derived contaminants were observed only in a limited number of samples, including Jar (0.561%) and Manufactura shower (2.152%). PP-derived contaminants were also detected at low levels in only a few samples, including Vanish Oxi (0.190%), Persil (0.380%), and Nivea shower (0.024%). Additional external contamination was identified in selected samples, including Feeleco (0.494%), Denk mit Color (0.081%), Tesori d’Oriente (0.791%), and Omeprazole (0.100%).

In summary, external contaminants contributed fewer compounds to the total number of identified compounds than polymer formulation and degradation-related compounds, and their occurrence showed high variability across samples. A comprehensive overview of external contamination compounds, including polystyrene (PS)-derived compounds, fluoropolymer-related surface residues, polypropylene (PP)-derived contaminants, and other external inputs, together with their occurrence across the analysed samples and associated statistical parameters, is provided in Supplementary Table S4. This pattern reflects the heterogeneous origin of external contamination within recycling streams.

### 3.6. Functional Relevance of Identified Compounds

To further organise the identified compounds, they were classified into three groups based on their functional relevance: (i) degradation-related compounds associated with structural changes of the polymer matrix; (ii) additive-derived transformation products contributing to the pool of non-intentionally added substances (NIAS), and (iii) persistent compounds detected across multiple samples.

Degradation-related compounds, including oxidation products and radical rearrangement products, were consistently detected across samples and serve as markers of polymer structural modification. Additive-derived compounds, such as phenolic structures, biphenyls, and oxirane-containing species, were also identified and correspond to transformation products of stabilisers and processing additives. A third group of compounds, including fluoropolymer-derived residues, long-chain alcohols, and wax-like hydrocarbons, was detected across multiple samples and represents compounds with persistent occurrence within the dataset.

### 3.7. Principal Component Analysis

Principal component analysis (PCA) was performed on aggregated chemical groups to explore relationships between degradation processes, additive-derived compounds, and contaminant profiles. The first two principal components explained 41.9% and 24.1% of the total variance, respectively. The PCA score plot showed a heterogeneous distribution of samples, with no distinct clustering by product category. Instead, samples were distributed along continuous gradients reflecting varying contributions of product-related inputs, fluoropolymer-derived compounds, degradation-related compounds, and additive-derived substances.

**Figure 8:**
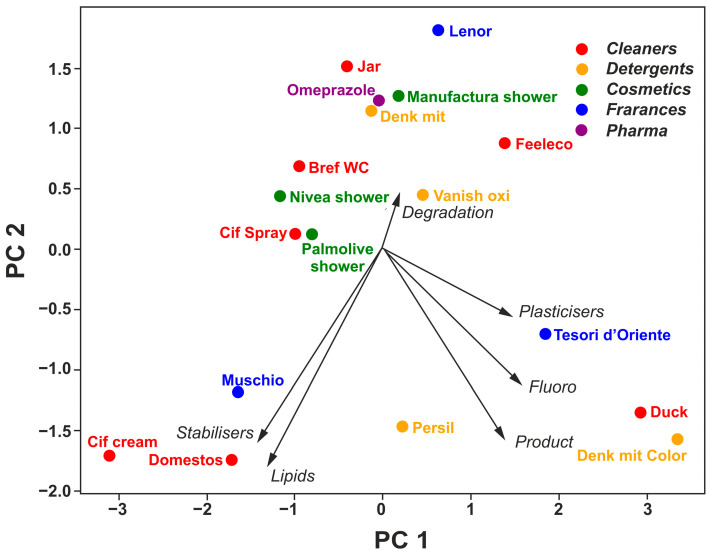
Principal component analysis (PCA) of aggregated chemical groups in rHDPE samples. Samples are colour-coded by product category; loading vectors indicate contributions of individual groups.

Samples such as Denk mit Color, Duck, and Tesori d’Oriente were positioned at higher values of PC1, corresponding to increased contributions of product-related and persistent compounds. In contrast, samples such as Cif cream and Domestos were located at lower PC1 values (Figure 8).

The second principal component (PC2) separated samples based on the relative contribution of stabiliser-related compounds and degradation-related compounds. Samples such as Lenor and Jar showed higher contributions of additive-derived compounds, whereas Persil and Duck showed higher contributions of degradation-related compounds. No distinct grouping of samples according to product category was observed.

## 4. Discussion

The present study provides a comprehensive chemical characterisation of post-consumer HDPE packaging, revealing a complex mixture of degradation products, additive-derived compounds, and external contaminants. The results highlight that the chemical composition of recycled HDPE is related to multiple overlapping factors, including material processing history, product contact, and cumulative transformation processes during use and recycling.

### 4.1. Polymer vs. Product Contributions

Product-related inputs can influence the chemical composition and properties of recycled HDPE; however, their effects are variable and strongly dependent on both material history and processing conditions [22]. The pharmaceutical packaging sample (Omeprazole), representing a system without lipid-rich product content, provides an important reference point for interpreting the origin of lipid-like compounds. Despite the absence of lipid-based formulation components, this sample exhibited a measurable contribution of compounds classified as having an ambiguous origin (Section 3.2; Figure 3b).

This observation indicates that a substantial fraction of lipid-like species originates from polymer-related components, such as waxes, slip agents, processing additives, or residual oligomeric structures, rather than product-derived residues. These ambiguous lipid-like compounds were consistently detected across all product categories (Figure 2b and Supplementary Figure S1), suggesting a systematic contribution from the polymer matrix itself. No correlation was observed between these compounds and polymer degradation products (Figure 5b), highlighting their independence from oxidation or radical-driven pathways.

Long-chain alcohols, including triacontanol, are not commonly used as functional plastic additives, but are known constituents of natural waxes and polymer-associated materials. While such compounds may occasionally occur in cosmetic formulations [23], they are not typical in detergents or most other products analysed in this study. Thermal and catalytic pyrolysis of polyolefins has been shown to produce similar long-chain oxygenated compounds and wax-like fractions [24,25], reinforcing a polymer-related origin.

In addition to lipid-like compounds, non-lipid product-derived compounds, including fragrance- and aroma-related substances, were identified in 15 of the analysed packaging samples (Section 3.2; Figure 3b), with the highest relative abundance observed in Jar (1.3%) and Manufactura shower (1.44%). m-Menthane derivatives appeared in five samples. These compounds interact with the polymer matrix through sorption and partitioning, enabling their persistence after product removal [26,27,28]. Their heterogeneous distribution, including absence in selected packaging types such as pharmaceutical samples, highlights their product-specific nature and distinguishes them from polymer-intrinsic compounds.

Together, these observations reveal two fundamentally different behaviours: (i) fragrance-related compounds primarily serve as markers of product-derived inputs, while (ii) lipid-like compounds with ambiguous origin predominantly reflect intrinsic polymer-derived contributions. Recognising this distinction is critical for interpreting chemical profiles and assessing the relevance of detected compounds for recycling quality (Figure 2 and Figure 3; Section 3.2, Section 3.3, Section 3.4 and Section 3.5).

In summary, this analysis demonstrates that the chemical profile of post-consumer HDPE is composed of both product residues and contributions from the polymer matrix. While fragrance and aroma compounds provide information about product-specific contamination, lipid-like compounds with an ambiguous origin indicate persistent polymer-related additives or processing residues, which are relevant for evaluating recyclability and potential impacts on material quality.

### 4.2. Stabilisers and Additive-Related Compounds

Stabilisers and additive-derived compounds represent a critical component of polyethylene packaging, as they are intentionally incorporated to prevent degradation but are themselves susceptible to transformation during processing and use. In the present study, stabiliser-related compounds were detected as both residual parent structures and transformation products, reflecting different stages of additive degradation (Section 3.3; Figure 4).

Most samples contained either stabiliser-related core compounds or their transformation products, but rarely both simultaneously, suggesting progressive conversion of stabilisers during the material lifecycle. This uneven distribution across samples (Figure 4) indicates that additive stability is strongly dependent on processing history and exposure conditions.

Identified compounds, including substituted phenols, biphenyls, and terphenyl derivatives, are consistent with degradation pathways of hindered phenolic antioxidants [29,30]. These compounds are formed through thermo-oxidative processes involving radical reactions between stabilisers and polymer-derived species [31]. Their presence across multiple samples (Section 3.3) confirms that stabiliser degradation is a common process in post-consumer HDPE.

Epoxide-containing compounds (oxiranes) were also detected and are associated with epoxidised lipid-based additives [32]. Their co-occurrence with long-chain alcohols and wax-like hydrocarbons (Section 3.3; Figure 4) suggests a shared origin related to polymer additives and processing aids [33,34]. These findings indicate interconnected transformation pathways linking stabiliser degradation and lipid-based additive chemistry.

**Figure 9:**
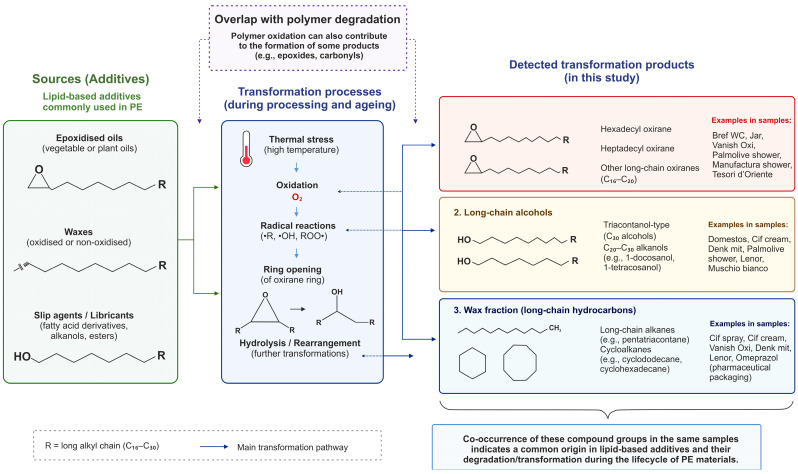
Proposed transformation pathways of stabilisers and lipid-based additives in polyethylene packaging.

The proposed transformation pathways are summarised in Figure 9, where phenolic antioxidants undergo thermo-oxidative reactions leading to substituted phenols and aromatic structures, while epoxidised additives generate oxirane-containing compounds. The simultaneous occurrence of these compounds, together with long-chain alcohols and wax-like hydrocarbons, reflects overlapping contributions from additive degradation and polymer transformation processes. At the same time, polyethylene itself can be transformed into wax-like materials during thermal and mechanical processing [35]. This further complicates the interpretation of lipid-like compounds, as similar structures may originate from both additive systems and polymer degradation.

The formation of these transformation products, particularly phenolic derivatives originating from stabilisers and epoxide-containing compounds associated with lipid-based additives, contributes to the pool of non-intentionally added substances (NIAS), which are increasingly recognised as relevant for the safety assessment of plastic materials [36]. Their structural diversity and co-occurrence across samples (Section 3.3) indicate the growing chemical complexity of polyethylene during its lifecycle.

In addition, these compounds may exhibit the potential to migrate from polymer matrices, further increasing their relevance in the context of material safety and regulatory evaluation [37]. While their concentrations in individual samples were generally low, their presence across multiple packaging types indicates that additive-derived NIAS represent a systematic component of recycled HDPE rather than isolated occurrences.

In summary, these results demonstrate that stabilisers and additive-derived compounds contribute not only to polymer protection but also to the evolving chemical complexity of recycled materials. Their transformation during processing results in structurally diverse compounds that persist in the material and must be considered when evaluating both recycling quality and chemical safety.

### 4.3. Polymer Degradation Processes

Polyethylene degradation is a complex, multi-step process involving several concurrent pathways, including primary oxidation, secondary oxidation, radical-driven chain scission and recombination, thermal degradation, and, under extreme conditions, aromatisation [21,38,39]. These processes are strongly influenced by thermal history, oxygen availability, mechanical stress, and environmental ageing during processing and recycling.

Within this framework, the degradation patterns observed in this study (Section 3.4; Figure 5) indicate that the chemical profile of the analysed rHDPE packaging materials is not primarily controlled by simple oxidative processes, but rather by advanced radical-driven transformation pathways associated with repeated thermal and mechanical processing.

The identification of primary and secondary oxidation products provides important insight into the early stages of polyethylene degradation. Primary oxidation in polyolefins is typically associated with the formation of hydroperoxides, alcohols, and carbonyl-containing compounds, which arise from the initial reaction of polymer chains with oxygen. These intermediates are inherently unstable and act as key precursors for further degradation reactions. Upon thermal or mechanical activation, hydroperoxides decompose into a range of secondary oxidation products, including aldehydes, ketones, and carboxylic acids. These compounds are widely recognised as characteristic markers of oxidative degradation in polyethylene and have been consistently reported in studies of thermo-oxidative degradation of HDPE [40,41]. In particular, low-molecular-weight aldehydes and ketones are typical secondary oxidation products formed by the decomposition of oxidised polymer segments. In the present study, the relatively low abundance of these compounds (Section 3.4; Figure 5) suggests that they are rapidly transformed into more stable structures, consistent with the fast progression of oxidative degradation reactions reported for polyethylene systems [41].

Instead of accumulating, these oxidation products appear to evolve into more stable degradation structures, as reflected by the dominance of radical rearrangement and cyclisation products. This subgroup, which includes cycloalkanes, cycloalkenes, and substituted cyclic hydrocarbons (e.g., 1-methyl-3-propylcyclohexane, butylcyclohexane, and (Z)-cycloundec-1-ene), represented the most abundant and diverse class of degradation-related compounds (n = 29; Section 3.4). These structures are characteristic of chain scission followed by radical recombination processes. Such pathways are known to dominate during repeated extrusion and mechanical recycling of polyethylene, where cumulative processing leads to progressive structural transformation of the polymer matrix [21,39]. The prevalence of these compounds in the analysed samples (Section 3.4; Figure 5) therefore indicates that the materials have undergone multiple thermal histories rather than a single degradation event. In contrast, the limited presence of thermal degradation products and aromatisation products provides additional insight into the degradation conditions. Aromatic hydrocarbons, including polycyclic aromatic hydrocarbons (PAHs), are typically associated with high-temperature pyrolytic processes [42]. Their low abundance in the analysed samples (Section 3.4; Figure 5) indicates that such extreme conditions were not dominant, and that the observed degradation pathways are more consistent with thermo-oxidative ageing and mechanical processing.

The observed chemical complexity of degradation products is further consistent with recent studies that highlight how plastic materials evolve into highly complex chemical systems over time due to the combined effects of degradation, additive transformation, and oligomer formation [43,44]. These accumulated compounds significantly contribute to the amount of non-intentionally added substances (NIAS), which represent a major issue in the evaluation of recycled plastics [45].

The degradation index (DI) introduced in this study provides a semi-quantitative measure of degradation intensity by integrating contributions from different degradation pathways (Section Degradation Index; Figure 6). The observed variability in DI values across samples shows that degradation is not uniform but strongly dependent on material history. Samples with elevated contributions of radical rearrangement products consistently exhibited higher DI values. In particular, detergent-related samples such as Denk mit and Vanish Oxi showed the highest DI values, whereas the pharmaceutical packaging sample (Omeprazole) exhibited the lowest DI, reflecting minimal degradation. This confirms that the DI captures differences in the relative contribution of degradation-related pathways rather than being solely driven by total compound abundance.

In summary, the results indicate that the degradation of compounds in rHDPE packaging materials is a multi-step process, with initial oxidation followed by extensive radical-driven transformation, ultimately leading to the formation of stable, structurally complex compounds. Importantly, the detected chemical profile reflects not only degradation processes but also the cumulative processing history of the material, which is essential for interpreting chemical composition and assessing recycling quality.

### 4.4. External Contamination and Fluoropolymer-Derived Compounds

External contamination represented the least diverse group of compounds identified in this study, yet it showed clearly sample-specific patterns (Section 3.1). Within this category, four subgroups were distinguished: polystyrene (PS)-derived compounds, polypropylene (PP)-derived compounds, fluoropolymer-derived residues, and other minor external contaminants. PS- and PP-derived compounds were detected sporadically and at low levels across the analysed samples (Figure 7a), consistent with incidental contamination from mixed polymer streams during collection and recycling, as reported in previous studies [46].

In contrast, fluoropolymer-derived compounds were detected across the majority of samples (Figure 7), highlighting their systematic occurrence. No correlation was observed between fluoropolymer residues and polymer degradation markers (Section 3.4), indicating that their presence is independent of thermo-oxidative or radical-driven degradation pathways. Instead, their distribution is governed by migration and surface accumulation, reflecting processing history and material mixing rather than intrinsic polymer changes.

Fluorinated compounds in HDPE can originate from both external contamination and intentionally added processing aids. Surface fluorination is commonly applied to improve chemical resistance, barrier properties, and wettability of polymer materials [47], while fluoropolymer-based processing aids, often combined with polyethylene glycol (PEG), are incorporated to enhance melt flow and reduce friction during extrusion [48]. These additives tend to migrate toward the polymer surface, forming thin fluorinated layers rather than distributing homogeneously.

Due to their chemical stability and resistance to degradation [49,50,51], fluoropolymer-derived residues persist through multiple recycling cycles, accumulating at material surfaces or within the bulk of recycled streams (Figure 7b). The variable abundance of selected fluorinated compounds, including dotriacontyl heptafluorobutyrate, heptadecyl heptafluorobutyrate, and octatriacontyl pentafluoropropionate, underscores differences in sample history and product category. Their co-occurrence with other halogenated hydrocarbons indicates multiple sources of external contamination contributing to the chemical complexity of post-consumer HDPE.

In summary, external contamination, and particularly fluoropolymer residues, represents a complementary dimension to polymer degradation and additive transformation in determining the chemical profile of recycled HDPE. Their persistence across most samples (Figure 7) reinforces the need for chemistry-informed sorting strategies that account not only for polymer composition but also for surface-derived and persistent contaminants.

### 4.5. Linking Degradation Pathways and Contaminant Profiles

**Figure 10:**
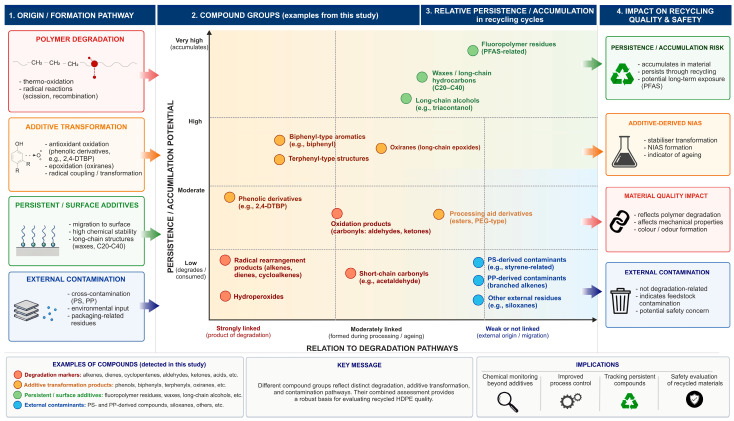
Conceptual framework linking degradation pathways and contaminant profiles in post-consumer HDPE. The figure illustrates the relationships between major compound groups, including polymer degradation products, additive-derived transformation products, persistent compounds, and external contamination, and their combined contribution to the chemical composition of recycled HDPE.

The relationships between degradation pathways, additive transformation, and contaminant profiles, as observed in Section 3.3, Section 3.4, Section 3.5, Section 3.6 and Section 3.7 (Figure 4, Figure 5, Figure 6, Figure 7 and Figure 8), are summarised in a conceptual framework (Figure 10), which integrates the identified compound groups and provides a mechanistic interpretation of how different chemical processes jointly determine the composition of post-consumer HDPE.

The results demonstrate that the analysed samples are not structured according to product category but rather along gradients defined by degradation processes, additive-derived compounds, and persistent contaminants (Section 3.7; Figure 8). This result highlights that the chemical composition of recycled HDPE is governed by multiple interacting factors rather than a single dominant source, reflecting the cumulative material history.

Within this framework, degradation-derived compounds, particularly radical rearrangement and cyclisation products, represent the dominant and most diverse subgroup (Section 3.4; Figure 5), indicating advanced structural transformation of the polymer matrix. Samples with higher contributions of these compounds exhibited elevated degradation index (DI) values (Section Degradation Index; Figure 6), confirming that DI reflects the progression toward more advanced degradation pathways rather than total compound abundance alone. In contrast, primary and secondary oxidation products were present at lower levels, suggesting that early-stage oxidation is rapidly followed by further transformation into more stable structures.

In parallel, additive-derived transformation products, including phenolic derivatives and oxirane-containing compounds (Section 3.3; Figure 4), contribute to the pool of non-intentionally added substances (NIAS) and reflect the progressive transformation of stabilisers and processing additives. At the same time, persistent compounds, such as fluoropolymer-derived residues and polymer-related lipid structures (Section 3.3 and Section 3.5; Figure 7), were detected across multiple samples, indicating accumulation processes largely independent of degradation.

The combined occurrence of these compound groups demonstrates that the chemical composition of recycled HDPE reflects not a single process but the interaction of multiple pathways. Degradation processes determine the structural modification of the polymer, additive transformation contributes to chemical complexity, and persistent compounds reflect material mixing and processing history.

Importantly, the absence of clustering according to product category (Figure 8) further supports the conclusion that chemical composition is governed by cumulative processing and use history rather than initial application. This integrated perspective provides a more realistic representation of post-consumer plastics compared to controlled laboratory studies. To further operationalise the conceptual framework, the analysed samples were classified according to their dominant chemical profiles, reflecting degradation impact, additive-derived transformation, persistence, and external contamination (Table 1). This classification links the conceptual framework to individual materials. It demonstrates how different chemical processes combine within specific samples and provides a practical interpretation of the variability observed across the dataset.

In summary, the conceptual framework presented in Figure 10 highlights that recycling quality is controlled by the combined effects of degradation, additive transformation, and persistence of specific compound groups, providing a basis for interpreting complex chemical datasets in recycled polyolefins.

### 4.6. Implications for Recycling Quality

The identified compound groups can be interpreted as chemical indicators linking material history to recycling performance, providing a functional connection between chemical composition and material quality. Degradation markers, particularly radical rearrangement and cyclisation products, reflect structural modification of the polymer matrix and are associated with cumulative processing history (Section 3.4; Figure 5). Their relationship with elevated degradation index (DI) values (Section Degradation Index; Figure 6) further confirms their relevance as indicators of advanced degradation rather than total compound abundance.

The absence of clustering in the PCA analysis (Section 3.7; Figure 8) demonstrates that the chemical composition of recycled HDPE is not determined solely by product category, but rather by a combination of degradation processes, additive transformation, and contamination pathways. This highlights the limitation of conventional sorting strategies based only on material type and underscores the importance of considering chemical composition in recycling systems.

From a practical perspective, these findings support the integration of chemical profiling into recycling quality assessment. Monitoring selected marker compounds, such as radical rearrangement products, phenolic derivatives, aromatisation-related structures, and fluorinated residues, provides a feasible approach for evaluating chemical characteristics relevant to recycled HDPE. The variability observed across samples (Section 3.2, Section 3.3, Section 3.4 and Section 3.5; Figure 3, Figure 4, Figure 5, Figure 6 and Figure 7) further suggests that threshold-based approaches for identifying materials less suitable for high-quality recycling are conceptually achievable.

Based on the observed chemical patterns, a set of indicative compound groups can be proposed for the rapid screening of recycled HDPE. Radical rearrangement products (e.g., substituted cycloalkanes and cycloalkenes) reflect advanced polymer degradation, while phenolic derivatives indicate stabiliser transformation and antioxidant depletion. Aromatisation-derived compounds, including PAHs and related condensed structures, represent advanced degradation pathways associated with radical cyclisation and condensation processes. In addition, fluorinated compounds can serve as markers of external contamination originating from processing aids or contact materials.

In this context, recycling quality is interpreted in terms of chemical integrity and contamination level rather than directly measured material performance. The applied Py-GC/MS approach provides detailed information on degradation pathways, additive transformation, and the presence of contaminants; however, it does not directly quantify mechanical, thermal, or rheological properties of the material.

Nevertheless, the identified chemical markers represent relevant indicators of structural modification of the polymer matrix and may serve as indirect proxies for degradation-related changes that can influence material performance. The proposed framework is therefore intended for comparative chemical assessment rather than direct prediction of functional material properties. In this regard, future studies may benefit from advanced experimental approaches, such as the use of isotope-labelled compounds, to enable more precise tracking of specific degradation pathways and transformation mechanisms in polymer systems.

## 5. Conclusions

This study provides a comprehensive chemical characterisation of post-consumer HDPE packaging materials. It demonstrates that their composition is formed by the combined effects of polymer degradation, additive transformation, and external contamination. Polymer degradation was identified as the dominant process shaping the chemical profile, with radical rearrangement and cyclisation products representing the most abundant and characteristic degradation markers. This indicates that repeated thermal and mechanical processing leads to advanced structural transformation of the polymer matrix, beyond simple oxidative degradation.

A key finding of this work is that lipid-like compounds, commonly attributed to product residues, are widely present even in samples with minimal product-derived input. This suggests that a substantial fraction of these compounds originates from the polymer matrix itself, highlighting the limitations of origin-based classification in recycled plastics. In addition, fluoropolymer-derived residues were detected across the majority of samples and showed no correlation with degradation processes, suggesting that they represent a distinct class of persistent contaminants originating from external inputs or processing-related sources rather than from polymer degradation.

The absence of clustering by product category confirms that the chemical composition of recycled HDPE is not directly determined by the original application but rather by a combination of degradation history, additive transformation, and contamination pathways. Overall, these findings demonstrate that degradation markers, additive-derived compounds, and persistent contaminants act as complementary indicators of material history and recycling quality. This supports the implementation of chemistry-informed recycling strategies, in which selected chemical markers are used for quality assessment, material selection, and process optimisation.

## Figures and Tables

**Table 1:** Functional classification of compound groups and their relevance for recycled HDPE.

Sample	Material Quality Impact (Degradation)	Additive-Derived Risk (NIAS)	Persistence/Accumulation Risk	External Contamination Risk
Duck	medium	medium	high (fluoropolymers + wax)	low
Domestos	medium	medium	high (fluoropolymers + lipids)	low
Feeleco	medium	low	high (fluoropolymers)	medium
Bref WC	medium–high	high (stabiliser residues)	medium	low
Jar	low–medium	high (core stabilisers)	low	low
Cif spray	medium	medium	medium	low
Cif cream	medium–high	medium	high (lipid + wax)	low
Vanish Oxi	high	medium	medium–high	low
Denk mit	high	medium	low–medium	low
Persil	medium	medium	medium	low
Denk mit Color	medium	medium	high (fluoropolymers)	medium
Palmolive shower	medium	medium–high	medium	low
Nivea shower	medium	high (aromatic additives)	medium	low
Manufactura shower	low–medium	high (mixed stabilisers)	low	medium
Tesori d’Oriente	low–medium	medium	high (fluoropolymers + wax)	medium
Muschio bianco	low	low	high (wax fraction)	low
Lenor	low	low	low	low
Omeprazole	very low	low	low	low

## Data Availability

The original contributions presented in this study are included in the article/Supplementary Material. Further inquiries can be directed to the corresponding author.

## Supplementary Materials

The following supporting information can be downloaded at: DOI: 10.5281/zenodo.19884847 and https://www.mdpi.com/article/10.3390/polym18111369/s1, Table S1: Product-related input compounds identified in post-consumer HDPE, including classification, occurrence across samples, and statistical parameters; Table S2: Polymer formulation-related compounds (stabilisers, lipid additives, and plasticisers) identified in post-consumer HDPE, including classification, occurrence across samples, and statistical parameters; Table S3: Polymer degradation-related compounds identified in post-consumer HDPE, including classification into oxidation, radical rearrangement, thermal degradation, and aromatisation products, with occurrence across samples and statistical parameters; Table S4: External contamination compounds identified in post-consumer HDPE, including PS-, PP-, fluoropolymer-derived, and other external inputs, with occurrence across samples and statistical parameters; Figure S1: Heatmap showing the distribution of compound subgroups across individual samples, based on the number of unique compounds in each subgroup. References [52,53,54,55,56,57,58,59,60,61,62,63,64] are cited in the supplementary materials.

## Abbreviations

The following abbreviations are used in this manuscript:

HDPE	High-Density Polyethylene
DI	Degradation Index
IUPAC	International Union of Pure and Applied Chemistry
NIAS	Non-Intentionally Added Substances
NIST	National Institute of Standards and Technology
PAHs	Polycyclic Aromatic Hydrocarbons
PC1	First Principal Component
PC2	Second Principal Component
PCA	Principal Component Analysis
PEG	Polyethylene Glycol
PP	Polypropylene
PS	Polystyrene
Py-GC/MS	Pyrolysis–Gas Chromatography–Mass Spectrometry
rHDPE	Recycled High-Density Polyethylene

## References

[1] Al-Salem S.M., Lettieri P., Baeyens J. (2009). Recycling and Recovery Routes of Plastic Solid Waste (PSW): A Review. Waste Manag..

[2] Ragaert K., Delva L., Van Geem K. (2017). Mechanical and Chemical Recycling of Solid Plastic Waste. Waste Manag..

[3] Geyer R., Jambeck J.R., Law K.L. (2017). Production, Use, and Fate of All Plastics Ever Made. Sci. Adv..

[4] Hopewell J., Dvorak R., Kosior E. (2009). Plastics Recycling: Challenges and Opportunities. Phil. Trans. R. Soc. B.

[5] Baser K.H.C., Buchbauer G. (2015). Handbook of Essential Oils: Science, Technology, and Applications.

[6] Burdock G.A. (2010). Fenaroli’s Handbook of Flavor Ingredients.

[7] Surburg H., Panten J. (2016). Common Fragrance and Flavor Materials: Preparation, Properties and Uses.

[8] Wypych G. (2025). Handbook of Polymers.

[9] Zweifel H., Amos S.E. (2001). Plastics Additives Handbook.

[10] Moldoveanu S.C. (2018). Pyrolysis of Organic Molecules: Applications to Health and Environmental Issues.

[11] Murat P., Harohalli Puttaswamy S., Ferret P.-J., Coslédan S., Simon V. (2020). Identification of Potential Extractables and Leachables in Cosmetic Plastic Packaging by Microchambers-Thermal Extraction and Pyrolysis-Gas Chromatography-Mass Spectrometry. Molecules.

[12] Pivnenko K., Eriksson E., Astrup T.F. (2015). Waste Paper for Recycling: Overview and Identification of Potentially Critical Substances. Waste Manag..

[13] Smith P., McLauchlin A., Franklin T., Yan P., Cunliffe E., Hasell T., Kurlin V., Kerr C., Attwood J., Shaver M.P. (2024). A Data-Driven Analysis of HDPE Post-Consumer Recyclate for Sustainable Bottle Packaging. Resour. Conserv. Recycl..

[14] Piringer O.G., Piringer O.G., Baner A.L. (2008). Plastic Packaging Materials for Food: Barrier Function, Mass Transport, Quality Assurance, and Legislation.

[15] Begley T., Castle L., Feigenbaum A., Franz R., Hinrichs K., Lickly T., Mercea P., Milana M., O’Brien A., Rebre S. (2005). Evaluation of Migration Models That Might Be Used in Support of Regulations for Food-Contact Plastics. Food Addit. Contam..

[16] Nerín C., Bourdoux S., Faust B., Gude T., Lesueur C., Simat T., Stoermer A., Van Hoek E., Oldring P. (2022). Guidance in Selecting Analytical Techniques for Identification and Quantification of Non-Intentionally Added Substances (NIAS) in Food Contact Materials (FCMS). Food Addit. Contam. Part A.

[17] Miralles P., Fuentes-Ferragud E., Socas-Hernández C., Coscollà C. (2025). Recent Trends and Challenges on the Non-Targeted Analysis and Risk Assessment of Migrant Non-Intentionally Added Substances from Plastic Food Contact Materials. Toxics.

[18] Celina M.C. (2013). Review of Polymer Oxidation and Its Relationship with Materials Performance and Lifetime Prediction. Polym. Degrad. Stab..

[19] Cuadri A.A., Martín-Alfonso J.E. (2017). The Effect of Thermal and Thermo-Oxidative Degradation Conditions on Rheological, Chemical and Thermal Properties of HDPE. Polym. Degrad. Stab..

[20] Chowreddy R.R., Fredriksen S.B., Anwar H., Mylvaganam B., Iveland A., Kamfjord T. (2026). Degradation Behaviour of Different Polyethylene and Polypropylene Materials under Long-Term Accelerated Weathering Conditions. J. Polym. Res..

[21] Patel A.D., Schyns Z.O.G., Franklin T.W., Shaver M.P. (2024). Defining Quality by Quantifying Degradation in the Mechanical Recycling of Polyethylene. Nat. Commun..

[22] Mylläri V., Hartikainen S., Poliakova V., Anderson R., Jönkkäri I., Pasanen P., Andersson M., Vuorinen J. (2016). Detergent Impurity Effect on Recycled HDPE: Properties after Repetitive Processing. J. Appl. Polym. Sci..

[23] Azmi N., Radzi S.M., Rehan M.M., Amin N.A.M. (2022). A Review on Cosmetic Formulations and Physicochemical Characteristics of Emollient and Day Cream Using Vegetable Based-Wax Ester. Malays. J. Sci. Health Technol..

[24] Yao L., Zhu J., Li S., Ma Y., Yue C. (2022). Analysis of Liquid Products and Mechanism of Thermal/Catalytic Pyrolysis of HDPE. J. Therm. Anal. Calorim..

[25] Wu J., Jiang Z., Cecon V.S., Curtzwiler G., Vorst K., Mavrikakis M., Huber G.W. (2024). The Effects of Polyolefin Structure and Source on Pyrolysis-Derived Plastic Oil Composition. Green Chem..

[26] Bai F., Chen G., Hu Y., Liu Y., Yang R., Liu J., Hou R., Li H., Wan X., Cai H. (2024). Understanding the Effect of Plastic Food Packaging Materials on Food Flavor: A Critical Review. Trends Food Sci. Technol..

[27] Kaur R., Kukkar D., Bhardwaj S.K., Kim K.-H., Deep A. (2018). Potential Use of Polymers and Their Complexes as Media for Storage and Delivery of Fragrances. J. Control. Release.

[28] Fuller J., White D., Yi H., Colley J., Vickery Z., Liu S. (2020). Analysis of Volatile Compounds Causing Undesirable Odors in a Polypropylene—High-Density Polyethylene Recycled Plastic Resin with Solid-Phase Microextraction. Chemosphere.

[29] Wiesinger H., Wang Z., Hellweg S. (2021). Deep Dive into Plastic Monomers, Additives, and Processing Aids. Environ. Sci. Technol..

[30] Costa J.P.D., Avellan A., Mouneyrac C., Duarte A., Rocha-Santos T. (2023). Plastic Additives and Microplastics as Emerging Contaminants: Mechanisms and Analytical Assessment. TrAC Trends Anal. Chem..

[31] Real L.E.P. (2023). Degradation and Stabilization of Polymers. Weathering of Polymers and Plastic Materials.

[32] Campanella A., Baltanás M.A. (2005). Degradation of the Oxirane Ring of Epoxidized Vegetable Oils with Hydrogen Peroxide Using an Ion Exchange Resin. Catal. Today.

[33] Jahan S., Rautela S., Chishti A.S., Shankhdhar D., Shankhdhar S.C., Srivastava A., Garg S.K. (2023). Triacontanol Is a Potent Alleviator of Stress Induced by Salt and Heavy Metal Contamination in Plants. Rhizosphere.

[34] Yetgin S., Gonen M., Savrik S.A., Balkose D., Barzic A.I., Rawat N.K., Haghi A.K. (2021). Polyethylene Wax: Uses, Characterization, and Identification. Imidic Polymers and Green Polymer Chemistry.

[35] Shaker M., Muzata T.S., Hamdani S.S., Wyman I., Saffron C.M., Rabnawaz M. (2024). Chemical Upcycling of High-Density Polyethylene into Upcycled Waxes as Rheology Modifiers and Paper Coating Materials. J. Clean. Prod..

[36] Kato L.S., Conte-Junior C.A. (2021). Safety of Plastic Food Packaging: The Challenges about Non-Intentionally Added Substances (NIAS) Discovery, Identification and Risk Assessment. Polymers.

[37] Remezov A., Knaup B., Wermann S., Marchesi D., Rüttler F., Vetter W. (2025). Factors Influencing Migration of NIAS: Model Experiments with 7,9-Di-Tert-Butyl-1-Oxaspiro(4,5)Deca-6,9-Diene-2,8-Dione (Arvin 8). Food Packag. Shelf Life.

[38] Ni Q., Yu F., Chen J., Xu L., Liu Z., Liu D., Jia H. (2026). Environmental Aging Effects on the Thermal Oxidative Degradation and Mechanical Failure of HDPE. J. Macromol. Sci. Part B.

[39] Felgel-Farnholz A., Schweighuber A., Klampfl C.W., Fischer J. (2023). Comparative Study on the Degradation of HDPE, LLDPE and LDPE during Multiple Extrusions. Polym. Degrad. Stab..

[40] Babetto A.S., Antunes M.C., Bettini S.H.P., Bonse B.C. (2020). A Recycling-Focused Assessment of the Oxidative Thermomechanical Degradation of HDPE Melt Containing pro-Oxidant. J. Polym. Env..

[41] ShahsavariBadvestani T., Jahanmardi R., Tayouri M., Fathi M. (2023). Acceleration of Thermo-Oxidative Degradation of High-Density Polyethylene Using Oxidized Polyethylene. Polyolefins J..

[42] Natesakhawat S., Weidman J., Garcia S., Means N.C., Wang P. (2024). Pyrolysis of High-Density Polyethylene: Degradation Behaviors, Kinetics, and Product Characteristics. J. Energy Inst..

[43] Law K.L., Sobkowicz M.J., Shaver M.P., Hahn M.E. (2024). Untangling the Chemical Complexity of Plastics to Improve Life Cycle Outcomes. Nat. Rev. Mater..

[44] Monclús L., Arp H.P.H., Groh K.J., Faltynkova A., Løseth M.E., Muncke J., Wang Z., Wolf R., Zimmermann L., Wagner M. (2025). Mapping the Chemical Complexity of Plastics. Nature.

[45] Horodytska O., Cabanes A., Fullana A. (2020). Non-Intentionally Added Substances (NIAS) in Recycled Plastics. Chemosphere.

[46] Brouwer M., Picuno C., Thoden Van Velzen E.U., Kuchta K., De Meester S., Ragaert K. (2019). The Impact of Collection Portfolio Expansion on Key Performance Indicators of the Dutch Recycling System for Post-Consumer Plastic Packaging Waste, a Comparison between 2014 and 2017. Waste Manag..

[47] Kharitonov A.P., Simbirtseva G.V., Tressaud A., Durand E., Labrugère C., Dubois M. (2014). Comparison of the Surface Modifications of Polymers Induced by Direct Fluorination and Rf-Plasma Using Fluorinated Gases. J. Fluor. Chem..

[48] Dehghani S. (2023). Mechanism of the Esterified Processing Aid Based on PEG and Fluoropolymers in Molten High-Density Polyethylene (HDPE). J. Polym. Res..

[49] Ebnesajjad S. (2013). Handbook of Biopolymers and Biodegradable Plastics: Properties, Processing and Applications.

[50] Hunt S.B., Román A.J., Wang X., Perez J.M., Perras F.A., Lee B., Xu J., Delferro M. (2025). Fluoropolymer Composites from Partially Perfluoroalkylated Waste Polyethylene. ACS Appl. Mater. Interfaces.

[51] Ebnesajjad S. (2015). Fluoroplastics.

[52] Lange B.M., Ahkami A. (2013). Metabolic Engineering of Plant Monoterpenes, Sesquiterpenes and Diterpenes—Current Status and Future Opportunities. Plant Biotechnol. J..

[53] Dudareva N., Negre F., Nagegowda D.A., Orlova I. (2006). Plant Volatiles: Recent Advances and Future Perspectives. Crit. Rev. Plant Sci..

[54] Ceretti D.V.A., Edeleva M., Cardon L., D’hooge D.R. (2023). Molecular Pathways for Polymer Degradation during Conventional Processing, Additive Manufacturing, and Mechanical Recycling. Molecules.

[55] Api A.M., Belsito D., Biserta S., Botelho D., Bruze M., Burton G.A., Buschmann J., Cancellieri M.A., Dagli M.L., Date M. (2021). RIFM Low-Exposure Fragrance Ingredients Safety Assessment. Food Chem. Toxicol..

[56] Barel A.O., Paye M., Maibach H.I. (2014). Handbook of Cosmetic Science and Technology.

[57] Wampler T.P. (2006). Applied Pyrolysis Handbook.

[58] Frankel E.N. (2014). Lipid Oxidation.

[59] Jetter R., Kunst L., Samuels A.L., Roberts J.A. (2018). Composition of Plant Cuticular Waxes. Annual Plant Reviews Online.

[60] Al-Malaika S., Issenhuth S. (1999). The Antioxidant Role of α-Tocopherol in Polymers III. Nature of Transformation Products during Polyolefins Extrusion. Polym. Degrad. Stab..

[61] Reingruber E., Himmelsbach M., Sauer C., Buchberger W. (2010). Identification of Degradation Products of Antioxidants in Polyolefins by Liquid Chromatography Combined with Atmospheric Pressure Photoionisation Mass Spectrometry. Polym. Degrad. Stab..

[62] Kissa E. (2001). Fluorinated Surfactants and Repellents.

[63] Carlsson D.J., Clark F.R.S., Wiles D.M. (1976). The Photo-Oxidation of Polypropylene Monofilaments: Part I: Chemical Changes and Mechanical Deterioration. Text. Res. J..

[64] Kimukai H., Kodera Y., Koizumi K., Okada M., Yamada K., Hiaki T., Saido K. (2020). Low Temperature Decomposition of Polystyrene. Appl. Sci..

