# Supplementary material for: Linking Degradation Pathways, Additive Transformation, and Contaminant Profiles in Post-Consumer HDPE: Implications for Recycling Quality
Source: Polymers (Basel). 2026 May 31;18(11):1369. doi: 10.3390/polym18111369 (PMC13259041; doi:10.3390/polym18111369)
Supplement: Supplementary file 1 [file polymers-18-01369-s001.zip › Supplementary material_ FigureS1.pdf]

Supplementary material – Figure S1

Linking degradation pathways, additive transformation, and contaminant profiles in post-consumer HDPE: implications for recycling quality

Marek Kucbel [1,*], Helena Raclavská [1], Jana Růžičková [1], Michal Šafář [1], Barbora Švédová [1], Karolina Slamová [2], Pavel Kantor [1] and Petr Braun [3]

[1] ENET Centre, CEET, VSB–Technical University of Ostrava, 17. listopadu 15/2172, 708 00 Ostrava-Poruba, Czech Republic; helena.raclavska@vsb.cz (H.R.); jana.ruzickova@vsb.cz (J.R.); michal.safar@vsb.cz (M.Š.); barbora.svedova@vsb.cz (B.Š.); pavel.kantor@vsb.cz (P.K.)
[2] Institute of Foreign Languages, VSB–Technical University of Ostrava, 17. listopadu 15/2172, 708 00 Ostrava-Poruba, Czech Republic; karolina.slamova@vsb.cz (K.S.)
[3] TESO, Urbánkova 3367, 143 00 Praha 4, Czech Republic; braun.teso@gmail.com (P.B.)
* Correspondence: marek.kucbel@vsb.cz (M.K.); Tel.: +420-596-995-448

Supplementary Figure S1: Heatmap showing the distribution of compound subgroups across individual samples, based on the number of unique compounds in each subgroup.

		Hard surface cleaners							Laumdry detergents				Cosmetic products			Fragrances / fabric softeners			Pharma-ceutical
		Duck	Domestos	Feeleco	Bref WC	Jar	Cif spray	Cif cream	Vanish oxi	Denk mit	Persil	Denk mit color	Palmolive shower	Nivea shower	Manufactura shower	Tesori d´ oriente	Muschio bianco	Lenor	Omeprazol
Product-related inputs	Non-lipid residues (fragrances, aromatics)	1	0	2	1	5	2	2	0	2	4	1	1	2	4	1	2	2	0
	Lipid-like (product-derived)	3	1	2	3	0	3	4	3	2	0	2	3	3	0	1	2	1	1
	Lipid-like (ambiguous origin)	4	1	3	3	1	3	4	7	5	5	6	4	6	1	3	5	2	4
Polymer formulation chemistry	Additives and stabilisers (total)	4	2	2	4	8	1	1	2	1	1	1	1	2	7	1	0	2	0
	Polymer-related lipids (total)	7	4	5	4	1	11	9	10	11	10	11	9	11	1	3	11	3	3
	Plasticisers and derived products	3	0	0	0	0	0	0	0	0	0	0	1	1	1	1	0	0	2
Polymer degradation processes	Primary oxidation products	1	4	0	0	0	0	0	0	1	0	1	1	1	1	1	1	1	0
	Secondary oxidation products	3	0	1	2	0	1	2	1	1	2	2	1	2	1	0	2	1	0
	Radical rearrangement and cyclisation products	13	0	12	11	0	16	13	16	15	4	13	19	12	0	14	14	4	11
	Thermal degradation products (alkenes, dienes)	0	0	0	0	0	0	0	0	1	1	1	1	1	1	1	1	1	1
	Aromatisation products (PAHs and related)	2	0	1	2	2	1	1	1	1	0	2	2	2	7	2	1	1	1
	Compounds of uncertain origin	0	0	0	0	1	0	1	0	1	1	1	1	0	1	0	0	2	0
	Additive-related transformation products	0	0	0	0	4	0	0	0	0	0	0	0	0	4	0	0	0	0
External contamination	Polystyrene (PS)-derived contaminants	0	0	0	0	7	0	0	0	0	0	0	0	0	8	0	0	0	0
	Fluoropolymer-derived surface residues	2	1	2	1	1	2	1	1	1	2	2	1	2	1	3	2	2	2
	Polypropylene (PP)-derived contaminants	0	0	0	0	0	0	0	2	0	1	0	0	1	0	0	0	0	0
	External contamination (incl. cross-polymer sources)	0	0	1	0	0	0	0	0	0	1	0	0	0	0	1	0	1	1

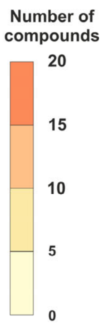

Total number of unique compounds across all samples: 137

Counting based on unique molecular identities. A compounds may occur in multiple samples but is counted once in each subgroup.
